# Weighted Score Tests Implementing Model-Averaging Schemes in Detection of Rare Variants in Case-Control Studies

**DOI:** 10.1371/journal.pone.0139355

**Published:** 2015-10-05

**Authors:** Brandon Coombes, Saonli Basu, Sharmistha Guha, Nicholas Schork

**Affiliations:** 1 Division of Biostatistics, School of Public Health, University of Minnesota, Minneapolis, MN, United States of America; 2 J. Craig Venter Institute, La Jolla, CA, United States of America; University of North Carolina, UNITED STATES

## Abstract

Multi-locus effect modeling is a powerful approach for detection of genes influencing a complex disease. Especially for rare variants, we need to analyze multiple variants together to achieve adequate power for detection. In this paper, we propose several parsimonious branching model techniques to assess the joint effect of a group of rare variants in a case-control study. These models implement a data reduction strategy within a likelihood framework and use a weighted score test to assess the statistical significance of the effect of the group of variants on the disease. The primary advantage of the proposed approach is that it performs model-averaging over a substantially smaller set of models supported by the data and thus gains power to detect multi-locus effects. We illustrate these proposed approaches on simulated and real data and study their performance compared to several existing rare variant detection approaches. The primary goal of this paper is to assess if there is any gain in power to detect association by averaging over a number of models instead of selecting the best model. Extensive simulations and real data application demonstrate the advantage the proposed approach in presence of causal variants with opposite directional effects along with a moderate number of null variants in linkage disequilibrium.

## Introduction

Genome-wide association studies (GWASs) have successfully identified many common genetic variants that are associated with a given outcome, but little risk can be explained by these identified single nucleotide polymorphisms (SNPs). There are several hypotheses for genetic factors contributing to disease risk [1–4]. One such hypothesis is that rare variants (RVs) measured in sequencing studies with large effect sizes contribute to the disease risk. However, the low minor allele frequency (MAF) of a RV makes it difficult to detect individual effects. Thus, rare-variant models are used to detect the combined effect of a set of RVs, such as the RVs within a candidate gene.

The existing approaches for rare variant detection can be broadly classified into three separate categories: (1) Collapsing methods based on pooling multiple RVs such as the Sum test [5], Cohort Allelic Sums Test (CAST) [6], Combined Multivariate and Collapsing (CMC) [7], Weighted Sum (W-Sum) test of Madsen and Browning [8], Kernel Based Adaptive Cluster (KBAC) [9], Replication Based Test (RBT) [10], ARIEL test [11], and EREC test [12]; (2) methods based on model selection such as Seq-aSum and Seq-aSum-VS approaches [13, 14], Variable Threshold Test (VT) [15], RARECOVER method [16], Selective grouping method [17], and Step-Up approach [18]; and (3) methods based on treating RV effects as random effects such as SSU approach [5], C-alpha test [19], and SKAT approach [20]. Basu and Pan [14] studied the performance of several of these multi-marker tests under a variety of disease models. The Sum test [5] was most powerful when there were n causal variants with effects in opposite directions and when there were few or no non-causal RVs; otherwise, it suffered from substantial loss of power. In the presence of opposite association directions and non-causal RVs, the SSU and SKAT tests performed better than the other tests. The model-selection approaches performed in the middle of random effect and collapsing methods. According to Basu and Pan [14], the model selection method, especially Seq-aSum-VS approach, performed very well when there were both protective and deleterious causal RVs and very few non-causal RVs, but the performance of Seq-aSum-VS approach was not very impressive in the presence of a moderate or large number of non-causal RVs. These and other findings [14] have led to combining the strengths of collapsing and random effect methods such as SKAT-O [21], Fisher method [22] and MiST [23] as discussed in a recent review [24]. Also, it was recently suggested that using SKAT in the presence of RVs and common variants (CVs) may be less optimal due to weighting RVs to have much more importance than CVs [25]. To overcome this, an upweighting of the CVs was implemented in SKAT-C [25].

While many improvements have been made in the random effects and collapsing methods literature, this paper takes a closer look at the methods based on model selection, especially the Seq-aSum and Seq-aSum-VS approaches. The Seq-aSum-VS approach classifies RVs based on the direction of association (‘+1’ for positive association, ‘-1’ for negative association and ‘0’ for no association) and implements a sequential variable selection scheme to select the best model for association between the SNP-set and the disease. The only difference between the Seq-aSum approach and the Seq-aSum-VS approach is that the variable selection (‘0’ allocation for a variant) is not implemented in the former. The Seq-aSum-VS approach starts with putting all the RVs in the ‘+1’ group and proceeds by moving each RV sequentially to the other two groups and assigns the allocation (‘+1’, ‘-1’, or ‘0’) with highest likelihood to the RV. The process of choosing the best model in Basu and Pan’s [14] method can be compared to a stepwise regression, where one may not always find the best model due to this selection scheme. This is especially true if a particular allocation results in a slightly higher likelihood over the other two allocations. In this case, choosing the allocation with highest likelihood for a SNP might not be optimal, rather it might be more efficient to allow multiple allocations for a RV and construct a test that takes into account multiple plausible models for the disease-RV association. Moreover, the performance of the sequential search is often dependent on the ordering of the variants in this search mechanism. A model-averaging approach could potentially reduce the dependency on the ordering of the variants in this sequential search.

Another issue to note here is that model selection approaches use dimension-reduction strategies to substantially reduce the number of parameters one would require to fit these large number of RVs. Hence, any model we can construct is never going to be the true model that generated the data we observe. In other words, the set of models is clearly misspecified, and model selection is best seen as a way of approximating, rather than identifying, full reality ([26], pp. 20–23). A model-averaging approach, on the other hand, could have an advantage over this model selection scheme. By averaging over a number of models, a model-averaging approach reduces the uncertainties associated with selection of models. However, averaging over a large number of models, especially the uninformative ones could cause loss in power. In addition, the approach could be too computationally intensive to be useful.

Model-averaging has been well studied in the prediction literature. One popular method is Bayesian model-averaging approach (BMA) [27–29] which provides guidelines for selecting a subset of plausible models, calculates the posterior probability that a predictor has an effect given disease status, and demonstrates the improved predictive performance of this model-averaging approach over a model selection approach. However, there is not much literature on the performance of model-averaging in testing the effect of a predictor. Moreover, due to the low occurrence of RVs within the case-control set, RVs would have to be considered in aggregate which complicates the implementation of a model-averaging scheme. Here we aim to compare the performance of a model-averaging approach in detection of RVs versus a model selection approach, where the models that contribute to the model-averaging approach are selected in a data-adaptive way.

This work proposes a data-adaptive model-averaging technique that addresses the limitation in the Seq-aSum-VS approach. Specifically, we allow selection of a set of potential models through our model selection scheme and use a weighted score test to detect association instead of choosing the best model. The rest of the paper is organized as follows. In the Methods section, we describe the several existing approaches and propose several alternative model-averaging schemes. In the Results section, we compare the proposed schemes with model selection approaches through extensive simulation studies and a real data example. We conclude with a short summary and discussion outlining a few future research topics.

## Materials and Methods

The purpose of this study is to develop methods to improve the power of detection of association between a trait and a group of RVs, for example, RVs in a sliding window or in a functional unit such as a gene. Although we have only considered binary traits here, our method and some of the other methods can be easily extended to other types of traits.

Assume there are *n* unrelated individuals, where *Y*_*i*_ = 0 for *n*_0_ controls and *Y*_*i*_ = 1 for *n*_1_ cases and *n*_0_+*n*_1_ = *n*. The *k* RVs are denoted by *X*_*ij*_, *j* = 1, …, *k* for *i* = 1, 2, …, *n*. The variables *X*_*ij*_ can take values such as 0, 1 and 2 corresponding to the number of minor alleles present. We do not take into account adjusting for covariates, such as environmental factors, though all of the methods are based on logistic regression and can accommodate covariates.

### 2.1 Existing Approaches

A logistic main effect model to test for association between a binary trait *Y*_*i*_ and *k* RVs is written as

$$\begin{array}{c} \text{Logit}\mathrm{Pr}\left(Y_{i}=1\right)=\beta_{0}+\sum_{j=1}^{k}X_{ij}\beta_{j};\;\;\;\;\;i=1,...,n. \end{array}$$ (1)

To test for association between the trait and these *k* RVs, the null hypothesis of no association is formulated as

$$\begin{array}{c} H_{0}:\beta ={\left(\beta_{1},...,\beta_{k}\right)}^{\prime }=0. \end{array}$$

One could perform a score test for the null hypothesis *H*_0_, but estimating the effect of an individual RV may not be feasible and the approach loses power in the presence of many null RVs. Hence, different techniques such as collapsing methods, random effect models, and model selection methods mentioned in the introduction have been proposed to handle these high-dimensional RVs.

Basu et. al. [13] and Basu and Pan [14] have proposed the Seq-aSum and Seq-aSum-VS approaches to incorporate model selection in constructing a test for association. These approaches attempt to sort the SNPs/RVs into one of three groups (null, causal, or protective). The general model for these approaches is based on the model suggested by Hoffman [18],

$$\begin{array}{c} \text{Logit}\mathrm{Pr}\left(Y_{i}=1\right)=\beta_{0}+\beta_{c}\sum_{j=1}^{k}X_{ij}\gamma_{j};\;\;\;\;\;i=1,...,n;\;\;\;\;j=1,...,k, \end{array}$$ (2)

with *γ*_*j*_ = *w*_*j*_ *s*_*j*_, where *w*_*j*_ is a weight assigned to RV *j*, *s*_*j*_ = 1 or −1 indicating whether the effect of RV *j* is positive or negative, and *s*_*j*_ = 0 indicating the exclusion of RV *j* from the model (i.e. the SNP is unlikely to be associated with the trait). There is literature on how to choose appropriate *w*_*j*_ for a specific problem and, if needed, it is not difficult to incorporate such weights into the methods we describe in this section. Here we assume *w*_*j*_ = 1 for all *j* = 1, 2, …, *k* in any subsequent analysis.

The null hypothesis to test if there is an association between the trait and the RVs boils down to *H*_0_ : *β*_*c*_ = 0. Seq-aSum and Seq-aSum-VS use the data to adaptively determine the optimal allocation. The Seq-aSum-VS test essentially proceeds through the following sequence of events.

1. Start with *s*_*j*_ = 1 for all *j*.
2. for *j* in 1:*k*
  - Find out the maximized likelihood (maximized over *β*_*c*_) corresponding to *s*_*j*_ = −1, 0, 1.
  - Set *s*_*j*_ to be the value that corresponds to the largest maximized likelihood among the three possible allocations (-1, 0, or 1).

The method selects a model from among 2*k* + 1 candidate models. Due to the sequential nature of the estimation, it is not guaranteed that the best model (model with the highest likelihood) will be chosen. Nevertheless, it avoids searching over 3^*k*^ possible models and thus gains power in many situations, especially for a large number of RVs.

The Seq-aSum-VS approach chooses the best model among 2*k* + 1 models and thus allows for only one allocation (*s*_*j*_ = 0, 1, -1) for each RV *j*, *j* = 1, 2, …, *k*. For a large number of neutral RVs, choosing the best model might not be an efficient way to detect association. A neutral RV *j* does not necessarily give highest likelihood at *s*_*j*_ = 0. For a given dataset, it could have a non-significant increase in the likelihood at allocation *s*_*j*_ = 1 or *s*_*j*_ = −1. Choosing the allocation that provides highest likelihood for such a SNP could affect the optimal assignment of the following RVs. It might be more efficient to allow multiple allocations for a RV instead of choosing the one with the highest likelihood and construct a test that takes into account multiple plausible models for the disease-RV association.

**Fig 1:**
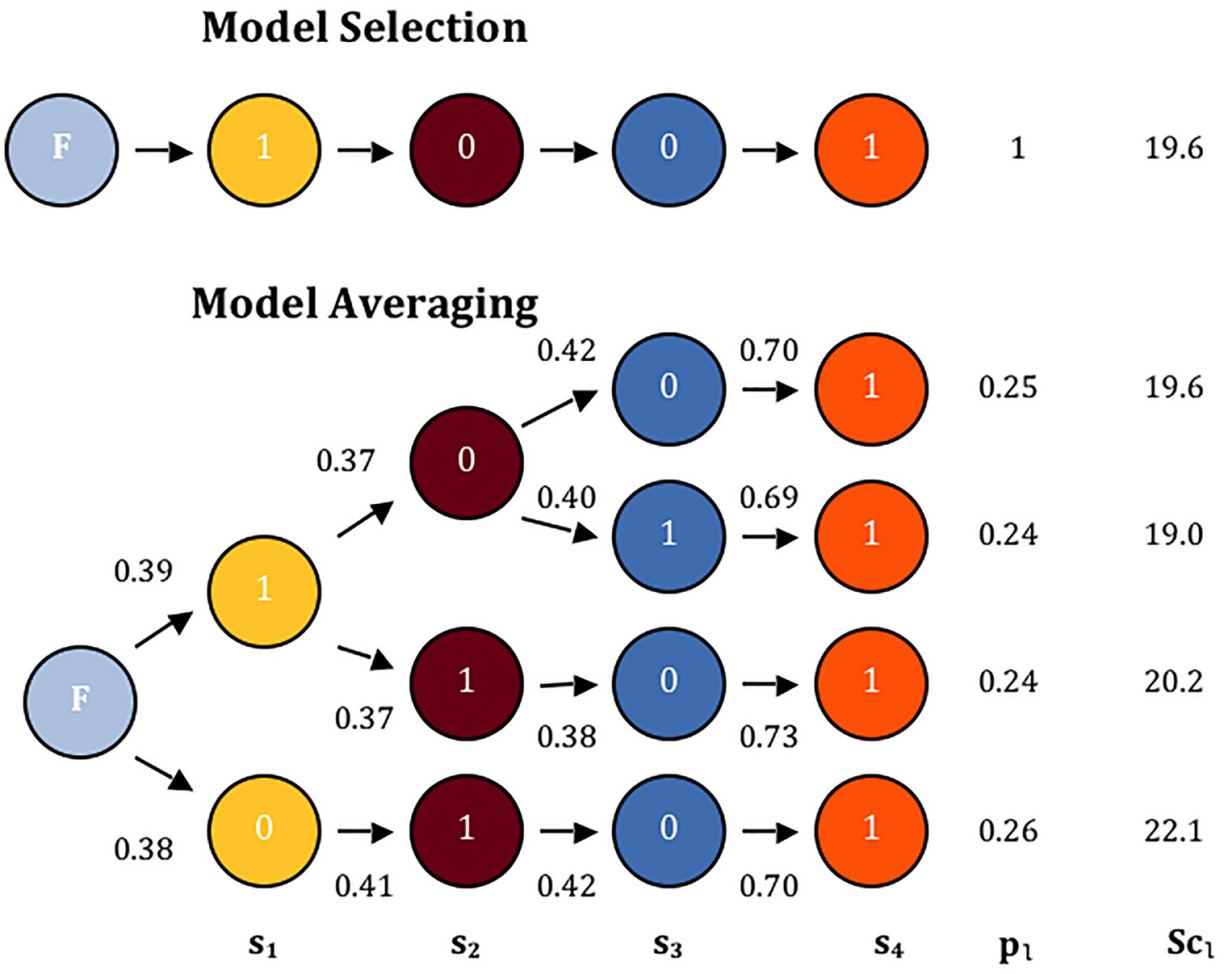
A demonstration of benefit of model-averaging (bottom) compared to model selection (top). The simulation setup is described in Section 3.3. F denotes the start of each algorithm where all RVs are allocated to being ‘positive’ group (*s*_*j*_ = 1 for all *j*). The numbers above the directional arrows on the model-averaging figure are ratios indicating how likely a given allocation at the current step should be selected. The paths with the highest ratio and any “close” ratios are explored. *p*_*l*_ is the path weight given by multiplying ratios along a path and scaling them so that the sum of path weights equals 1. Sc_*l*_ is the score statistic for each path (See Section 2.2 for details).

One such scenario where the model-averaging approach has advantage over the model-selection approach is where only the last RV is causal. Here, a sequential model selection algorithm could fail to find the best allocation due to the null RV diluting the effect of the lone causal RV [14]. For demonstration, we consider a scenario where out of 4 independent RVs in a set, only the last RV is causal. Fig 1 shows the paths taken for model selection (top) and model-averaging (bottom) for one such realization. By construction, model selection selects only one path. Model-averaging, however, computes how likely a given path is at each node and explores all likely paths. Section 2.2 explains one way to construct a measure to choose “likely” paths. In Fig 1, the model-averaging algorithm finds a better path (the bottom path) than the one selected by model selection. By averaging the four likely paths, we reduce the dependency on ordering and thus, gain power to detect association of the RVs with disease. In the next two subsections, we propose two path-finding algorithms to identify potential models to capture association between *k* RVs and the binary disease.

### 2.2 Model Branching through thresholding

This proposed approach provides a way to select multiple possible allocations (‘+1’,‘-1’ or ‘0’) for a RV. This method proceeds the same way as the Seq-aSum-VS approach. It starts with putting all the RVs in ‘+1’ group and proceeds by moving each RV sequentially to the other two groups (‘0’ or ‘-1’ group). The Seq-aSum-VS approach classifies the RV to the allocation with the highest likelihood, but the proposed approach here allows for multiple plausible allocations of a RV by selecting allocations that give high scores rather than just the highest score. If any two allocation scores are close, we explore both paths. The tree model proposed here proceeds through constructing a tree in the following way:

1. Set *s*_*j*_ = 1 for *j* = 1, …, *k*.
2. Choose a cutoff, *κ*, between 0 and 1 that determines if a score statistic is close to the highest score statistic.
3. Starting with *s*_1_, calculate the three score statistics corresponding to setting *s*_1_ = 1, 0 and −1. Denote them as *Sc*_1_, *Sc*_0_, and *Sc*_−1_, respectively. The score statistic *Sc*_*l*_ is given by
  $$\begin{array}{c} Sc_{l}=\frac{{\left[\sum_{i=1}^{n}\left(Y_{i}-\bar{Y}\right)\left(g_{i,l}-{\bar{g}}_{l}\right)\right]}^{2}}{\bar{Y}\left(1-\bar{Y}\right)\sum_{i=1}^{n}g_{i,l}^{2}}, \end{array}$$
  where $g_{i,l}=\sum_{j=1}^{k}X_{ij}s_{l}$ with *s*_*l*_ = 0, 1, or −1 and $s_{l^{\prime }}=1$ for all *l*′ ≠ *l*
4. Calculate the score ratio
  $$\begin{array}{c} R_{l}=\frac{Sc_{l}}{Sc_{1}+Sc_{0}+Sc_{-1}} \end{array}$$ (3)
  for *l* = 1, 0, and −1.
5. Allow allocations satisfying *R*_*l*_ ≥ max_*l*_{*R*_*l*_} × *κ*, similar to “Occam’s window” technique proposed by Madigan and Raftery [30]
6. For each allowed allocation of *s*_1_,
for *j* = 2:*k*
7. Repeat Steps 3-5 to find possible values for *s*_*j*_, *j* = 2, …, *k*.
8. Finally, obtain a tree, each branch of which represents a possible allocation for of (*s*_1_, …, *s*_*k*_).
9. For convenience, the weight of each branch *p*_*l*_ is calculated by taking the product of score ratios of successive branching steps.

Let there be finally *m* total branches, with overall weights *p*_1_, *p*_2_, …, *p*_*m*_ respectively. We propose a weighted score test using the above data adaptive model-averaging approach to test for the null hypothesis *H*_0_ in Section 2.4. We refer to this approach as Branching under Ratios (BUR) approach.

### 2.3 Selection of models using a weighted likelihood function

We also propose a model-averaging approach through a weighted likelihood function. In order to allow for multiple plausible allocations for a RV, we assume

$$\begin{array}{c} s_{j}=\{\begin{array}{cc} 1 & \text{with}\;\text{probability}\;\left(w.p.\right)\;\;q_{1}\\ 0 & w.p.\;\;q_{2}\\ -1 & w.p.\;\;1-q_{1}-q_{2} \end{array} \end{array}$$

where *j* = 1, 2, …, *k*. The tree model proposed in this article proceeds through constructing a tree in the following way

1. Set *s*_*j*_ = 1 for *j* = 1, …, *k*.
2. Choose a cutoff *κ* for between 0 and 1 for the branch probabilities.
3. Starting with *s*_1_, consider the likelihood of ***Y*** by averaging over the different possibilities of *s*_*j*_
  $$\begin{array}{c} f\left(Y|q_{1},q_{2},\beta_{0},\beta_{c}\right)=q_{1}L\left(\beta_{0},\beta_{c,1}\right)+q_{2}L\left(\beta_{0},\beta_{c,0}\right)+\left(1-q_{1}-q_{2}\right)L\left(\beta_{0},\beta_{c,-1}\right), \end{array}$$ (4)
  where for *h* = 1, 0, −1: $L(\beta_{0},\beta_{c,h})=\prod_{i=1}^{N}p_{h}^{Y_{i}}{\left(1-p_{h}\right)}^{Y_{i}}$ and $p_{1}=\frac{\text{exp}(\beta_{0}+\beta_{c,1}\sum_{j=1}^{k}X_{ij})}{1+\text{exp}(\beta_{0}+\beta_{c,1}\sum_{j=1}^{k}X_{ij})}$, $p_{0}=\frac{\text{exp}(\beta_{0}+\beta_{c,0}\sum_{j=2}^{k}X_{ij})}{1+\text{exp}(\beta_{0}+c\sum_{j=2}^{k}X_{ij})}$, and $p_{-1}=\frac{\text{exp}(\beta_{0}-\beta_{c,-1}X_{i1}+\beta_{c,-1}\sum_{j=2}^{k}X_{ij})}{1+\text{exp}(\beta_{0}-\beta_{c,-1}X_{i1}+\beta_{c,-1}\sum_{j=2}^{k}X_{ij})}.$
4. Maximize the likelihood in Eq (4) with respect to *q*_1_, *q*_2_, *β*_0_, *β*_*c*,1_, *β*_*c*,0_ and *β*_*c*, − 1_ and obtain the maximum likelihood estimates ${\hat{q}}_{1}$, ${\hat{q}}_{2}$, ${\hat{\beta }}_{0}$, ${\hat{\beta }}_{c,1}$, ${\hat{\beta }}_{c,0}$ and ${\hat{\beta }}_{c,-1}$ respectively.
5. Allow allocations satisfying a path probability greater than max{*q*_1_, *q*_2_, 1 − *q*_1_ − *q*_2_} × *κ*.
6. For each allowed allocation of *s*_1_,
for *j* = 2:*k*
7. Repeat Steps 3–5 to find possible values for *s*_*j*_, *j* = 2, …, *k*.
8. Finally, obtain a tree, each branch of which represents a possible allocation of (*s*_1_, …, *s*_*k*_).
9. Once again, the weight of each branch *p*_*l*_ is calculated by taking the product of $\hat{q}\text{s}$ in successive branching steps.

Let there be finally *m* total branches, with overall weights *p*_1_, *p*_2_, …, *p*_*m*_ respectively. We propose a weighted score test using the above data adaptive model-averaging approach to test for the null hypothesis *H*_0_ in Section 2.4. We will refer to this approach as Likelihood-based Model Branching (LiMB) method.

### 2.4 A weighted score test

We propose a weighted score test that computes score test statistics for testing *H*_0_ : *β*_*c*_ = 0 based on the model selected in each branch and subsequently averages all the score test statistics with their corresponding weights *p*_1_, …, *p*_*m*_ to compute the final weighted score test statistic. More precisely, let the model selected in the *l*-th branch be given by

$$\begin{array}{c} \text{Logit}\mathrm{Pr}\left(Y_{i}=1\right)=\beta_{0}+\beta_{c}g_{i}^{l},\;\;i=1,...,n;\;l=1,...,m, \end{array}$$ (5)

where $g_{i}^{l}=\sum_{j=1}^{k}X_{ij}s_{j}^{l}$ with $\left(s_{1}^{l},...,s_{k}^{l}\right)$, the allocation vector ***s*** selected in the *l*-th branch. After some routine algebra, the score test statistic for testing *H*_0_ : *β*_*c*_ = 0 corresponding to the *l*-th branch can be shown as

$$\begin{array}{c} Sc_{l}=\frac{{\left[\sum_{i=1}^{n}\left(Y_{i}-\bar{Y}\right)\left(g_{i}^{l}-{\bar{g}}_{l}\right)\right]}^{2}}{\bar{Y}\left(1-\bar{Y}\right)\sum_{i=1}^{n}{\left(g_{i}^{l}\right)}^{2}} \end{array}$$

The weighted score test denoted by *wscore* is defined as

$$\begin{array}{c} wscore=\sum_{l=1}^{m}Sc_{l}p_{l} \end{array}$$

The distribution of this data adaptive score test under the null hypothesis is not known, so one needs to use a permutation test or other simulation-based approach to derive a p-value for this weighted score test statistic *wscore*.

### 2.5 Paring the branches

For either pathfinding scheme, branch weights, *p*_1_, *p*_2_, ⋯, *p*_*m*_, are computed. Thus, we can further narrow the plausible models by choosing a cutoff, *q*_max_, such that selected branches have weights greater than or equal to *p*_max_ ⋅ *q*_max_. After paring our branches, we can recalculate the *wscore* for the best branches.

The simulation section described next discusses the advantages and tradeoffs of the *wscore* approach for each of the two different pathfinding approaches and their pared versions compared to model selection, collapsing, and random effects methods in terms of their power in a variety of simulation scenarios and a real data analysis.

## Results

### 3.1 Simulation study

In this section, the performance of the proposed weighted score tests implementing the model-averaging schemes is compared to the model selection approaches, such as Seq-aSum and Seq-aSum-VS described in section 2.1. We also compare these approaches to Sum test [5] and SKAT [20] with a weighted linear kernel. For this purpose, we simulate data as described in Basu and Pan [14]. In particular, we simulate *k* RVs each with MAF = 0.005 and each common variant (CV) with MAF = 0.2. To simulate the datasets, we generate a latent vector ***Z*** = (*Z*_1_, …, *Z*_*k*_)′ from a multivariate normal distribution with a first-order auto-regressive (AR1) covariance structure. There is a correlation *Corr*(*Z*_*i*_, *Z*_*j*_) = *ρ* ^∣*i* − *j*∣^ between any two latent components. For the purpose of this simulation, we have considered pairwise correlation of *ρ* = 0 and *ρ* = 0.9 which implies linkage equilibrium among the variants and strong linkage disequilibrium (LD) among the variants, respectively. Each component of the latent vector ***Z*** is then dichotomized to yield a haplotype, where the probability of ***Z*** being zero is the MAF corresponding to the RV. Next, we combine two independent haplotypes and obtain genotype data ***X***_*i*_ = (*X*_*i*1_, …, *X*_*ik*_)′. The disease status *Y*_*i*_ is then generated from a logistic regression model with or without interaction. We have considered a sample of 500 cases and 500 controls.

We consider several simulation set-ups. We first simulate 10000 datasets under the null hypothesis of no association between the variants and the disease. For every set of RVs or mix of CVs and RVs, we estimate the null distribution of the test statistics based on 10000 replicates and determine the 95th percentile of the null distribution for each test statistic. We next compare the power of all the competing methods based on 10,000 simulated datasets for a variety of situations. When available, the asymptotic power is used by determining the number of times the calculated test p-value is less than 0.05. Otherwise, the empirical power is determined by the number of times the test statistic was ≥ the 95th percentile determined from its null distribution.

We consider a variety of scenarios to test the performance of the proposed approaches. For demonstration, we first consider the situation displayed in Fig 1 where only the last RV is causal and the others are non-causal. Also, because Basu and Pan [14] concluded model selection methods were a good compromise for a vast number of situations whereas the random effects and collapsing methods performance depended heavily on directionality of association, we consider the following scenarios: (1) four RVs are causal and (2) two RVs are causal while two RVs are protective. Both no LD and strong LD are used in these situations. Lastly, we consider cases when there is a mix of CVs and RVs to further understand the real data results. For each model-averaging method in Section 2.2 and Section 2.3, *κ* must be chosen so that the number of paths does not become too large. While many *κ* satisfy this, we present the results on model-averaging-based tests after fixing *κ* at 0.90 for LiMB and 0.95 and 0.99 for BUR. We also select *q*_max_ = 0.99 for the pared version of our tests.

### 3.2 Simulation 1: Null distribution of the test statistic

**Fig 2:**
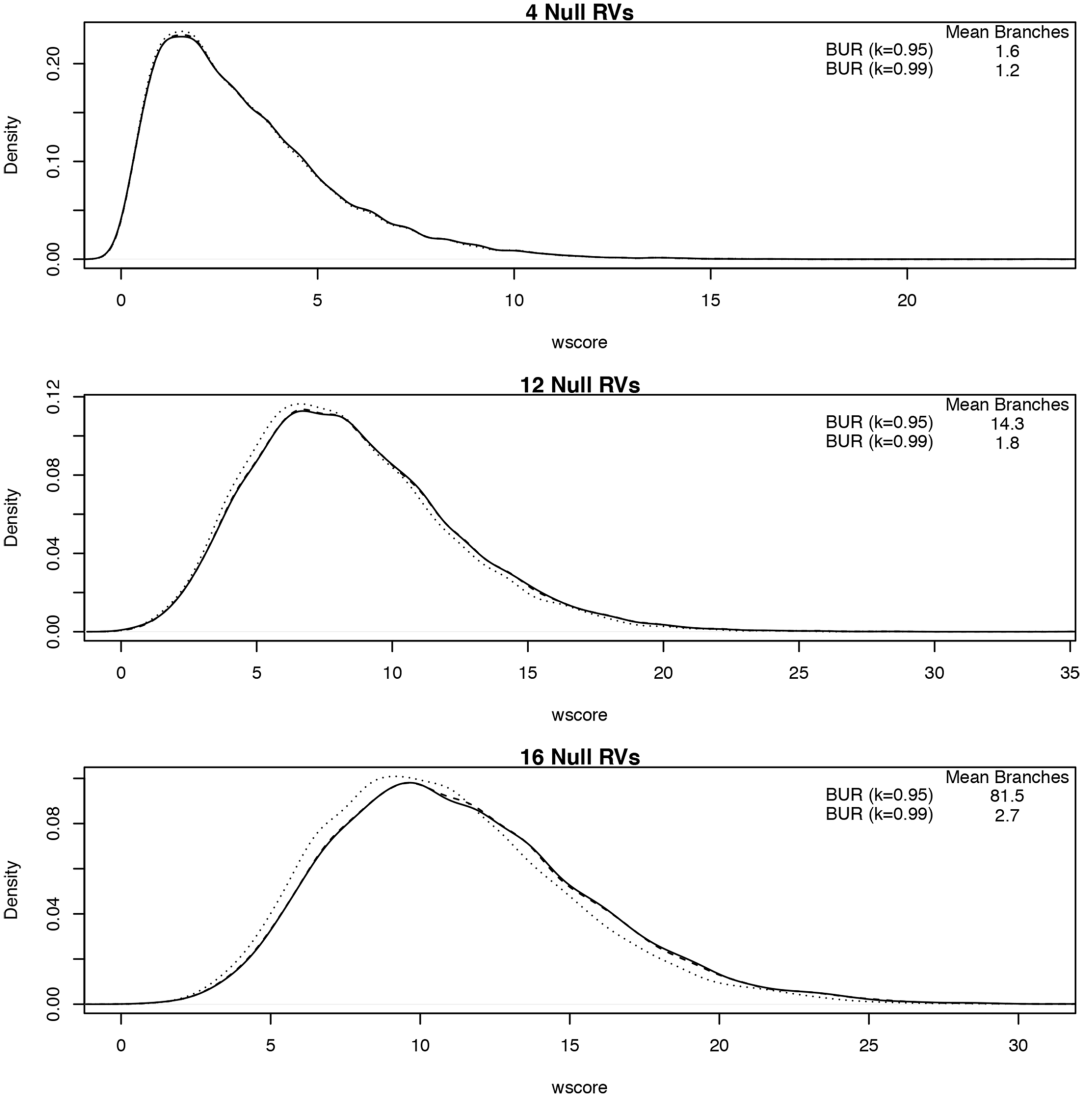
Density plot of model selection and BUR model-averaging approach test statistics under the null situation where all RVs are non-causal given a RV-set size of 4 (top), 16 (middle), or 28 (bottom). The x-axis is the value of the test statistic and the y-axis is the density of the distribution. Plotted in each figure are Seq-aSum-VS, BUR (*κ* = 0.95), and BUR (*κ* = 0.99) as solid, dotted, and dashed lines, respectively. The number of branches averaged over by these model-averaging approaches is in the upper right table of each plot.

Fig 2 shows the null density distribution of the test statistics of the BUR algorithm compared to Seq-aSum-VS while varying the number of null RVs included in the analysis. The average number of models averaged over are reported in the upper right corner. The wscore and Seq-aSum-VS statistics increase as the number of RVs increase and have the form of a mixture of *χ* ^2^ distributions. However, due to the data adaptive procedure and the varying number of models averaged over, we cannot derive the theoretical null distribution. By construction, if the *κ* for the BUR algorithm was set to 1, the *wscore* statistic would be exactly equal to Seq-aSum-VS. We can see that when the mean number of branches is small in Fig 2, the distributions are almost equivalent. As the number of RVs increases, the dotted line representing BUR with *κ* = 0.95, which has the most models averaged over, begins to move to the left of the Seq-aSum-VS distribution. In other words, Seq-aSum-VS becomes stochastically greater than BUR with *κ* = 0.95. The BUR with *κ* = 0.99 remains very close to the Seq-aSum-VS statistic for all cases displayed because the average number of models averaged over remains low.

### 3.3 Comparing power among different approaches

Next we compare the power of different approaches for rare variant detection under different alternative models.

#### Simulation 2: Effect of order dependency

Here, we demonstrate how model-averaging can address order dependency as in Fig 1. To do this, we set only the last RV to be causal with an odds ratio (OR) of 6. We then independently simulate 3, 7, 11, 15 and 19 null RVs in front of the causal RV. For each simulation setup we have generated 10000 replicates and have reported in Table 1 the empirical power of model selection methods (Seq-aSum, Seq-aSum-VS) and the direct model-averaging extension to Seq-aSum-VS, BUR, to demonstrate the reduction of order dependency by averaging over many models. The power is reported at a level of significance of 0.05. According to Table 1, as the number of null RVs before the one causal RV increases, the BUR (*κ* = 0.95) approach with pared branches becomes increasingly more powerful than Seq-aSum-VS. By exploring many paths and then excluding less likely paths, we reduce the dependency on the ordering of RVs and thus gain power to detect association here.

**Table 1:** (*α* = 0.05) Demonstration of how model-averaging can reduce path dependency. In this disease model, only the last RV in order is causal with OR = 6. Empirical power listed in the table based on 10000 replicates with a number of non-causal RVs before the causal RV. There is no LD among the RVs.

No. of non-causal RVs	3	7	11	15	19
Seq-aSum	0.897	0.740	0.644	0.561	0.465
Seq-aSum-VS	0.920	0.811	0.703	0.615	0.527
**BUR** (*κ* = 0.95)					
Average no. branches	2.2	6.3	31.4	222	2130
Average no. pared branches	1.2	1.4	1.8	2.4	3.3
wscore	0.919	0.808	0.692	0.593	0.504
pared wscore	0.914	0.811	0.710	0.631	0.539
**BUR** (*κ* = 0.99)					
Average no. branches	1.3	1.5	2.1	3.3	5.4
Average no. pared branches	1.2	1.1	1.1	1.2	1.4
wscore	0.920	0.812	0.703	0.617	0.527
pared wscore	0.919	0.812	0.707	0.618	0.536

#### Simulation 3: Power Comparison in presence of no LD

Here, we consider the situation where there is no LD between any two RVs, mimicking the situation where mutations are all completely random and independent of each other. Here we compare the power of our model-averaging approaches with several alternative methods. We first consider the situation where all 4 causal RVs share a common odds ratio (OR) of 2. We then simulate 0, 4, 8, and 12 null variants to study the impact of null variants on power. For each simulation setup we have generated 10000 replicates and reported the asymptotic or empirical power of a collapsing method (Sum), a random effect method (SKAT), model selection methods (Seq-aSum, Seq-aSum-VS), and model-averaging methods (BUR and LiMB) in Table 2.

As in Basu and Pan [14], we see that the Sum test performs well above the other methods when there are very few non-causal variants present. As we increase the number of non-causal RVs, SKAT obtains advantage over the Sum test. Model selection and model-averaging approaches obtain similar power to the collapsing approach as the number of non-causal RVs increases. Seq-aSum performs well when there is no null variant, but loses power compared to Seq-aSum-VS as in presence of null variants. The BUR (*κ* = 0.99) approach performs similarly to Seq-aSum-VS since only one or two paths are generally explored at *κ* = 0.99. The pared wcore for BUR (*κ* = 0.95) approach performs similar to the BUR (*κ* = 0.99) approach whereas BUR (*κ* = 0.95) loses little power due to averaging over too many null models. The LiMB approach does not perform well and has uniformly lower power than the other model averaging approaches.

**Table 2:** (*α* = 0.05) Independent RV analysis. Power in table based on 10000 replicates for each situation with a number of non-causal RVs.

	OR = (2,2,2,2)				OR = (4, 3, 1/3, 1/4)			
No. of non-causal RVs	0	4	8	12	0	4	8	12
Sum	0.710	0.482	0.362	0.289	0.501	0.315	0.237	0.191
SKAT	0.494	0.411	0.366	0.329	0.943	0.901	0.861	0.820
Seq-aSum	0.505	0.376	0.328	0.278	0.922	0.828	0.755	0.664
Seq-aSum-VS	0.500	0.397	0.337	0.285	0.906	0.836	0.768	0.681
**LiMB** (*κ* = 0.90)								
Average no. branches	1.5	2.2	3.8	6.9	1.1	1.7	2.7	4.5
Average no. pared branches	1.0	1.1	1.1	1.1	1.0	1.0	1.1	1.1
wscore	0.481	0.388	0.325	0.275	0.908	0.823	0.737	0.645
pared wscore	0.475	0.391	0.324	0.272	0.906	0.827	0.736	0.638
**BUR** (*κ* = 0.95)								
Average no. branches	1.4	3.4	16.4	97.0	1.5	4.2	18.5	121
Average no. pared branches	1.0	1.2	1.5	2.0	1.1	1.3	1.6	2.1
wscore	0.498	0.395	0.329	0.278	0.903	0.835	0.763	0.674
pared wscore	0.500	0.399	0.335	0.288	0.898	0.832	0.761	0.686
**BUR** (*κ* = 0.99)								
Average no. branches	1.1	1.4	1.9	2.8	1.1	1.4	1.9	2.9
Average no. pared branches	1.0	1.1	1.2	1.4	1.1	1.1	1.2	1.4
wscore	0.500	0.399	0.335	0.286	0.906	0.838	0.766	0.680
pared wscore	0.500	0.399	0.337	0.287	0.905	0.837	0.765	0.680

For the next scenario, the 4 causal RVs have various association strengths, OR = (4, 3, 1/3, 1/4). We then simulate 0, 4, 8, and 12 null variants to study the impact of null variants on power. Again for each simulation setup we have generated 10000 replicates and reported the asymptotic or empirical power of a collapsing method (Sum), a random effect method (SKAT), model selection methods (Seq-aSum, Seq-aSum-VS), and model-averaging methods (BUR and LiMB) in Table 2. As in Basu and Pan [14], SKAT performs best for any given number of non-causal RVs, while the Sum test suffers dramatic power loss. Model selection is only slightly less powerful than the random effect methods. Once again, the BUR approach performs similarly to Seq-aSum-VS when only one or two paths are explored, and the LiMB approach does not perform well. Overall, model-averaging methods do not show much advantage over model selection methods when there is no LD among the variants.

#### Simulation 4: Power Comparison in presence of LD

Table 3 considers the same cases to Table 2 except strong LD is present amongst the causal RVs and the non-causal RVs. When we have strong LD and causal RVs in the same direction, we can see that the collapsing and random effects methods perform similarly while model selection is lower powered. In this situation, there also appears to be no clear benefit to averaging over more models. This is because the non-causal RVs are strongly correlated with RVs that have effects in the same direction making them also have a marginal OR greater than 1. This means the best model is most likely one that is equivalent to the Sum test which is the first path that model selection and model-averaging considers. However, they are penalized by considering less likely models thereafter.

**Table 3:** (*α* = 0.05) RV analysis when there is strong LD among the RVs. Power in table based on 10000 replicates for each situation with a number of non-causal RVs.

	OR = (2,2,2,2)				OR = (4, 3, 1/3, 1/4)			
No. of non-causal RVs	0	4	8	12	0	4	8	12
Sum	0.999	0.971	0.904	0.838	0.263	0.287	0.296	0.284
SKAT	0.999	0.975	0.910	0.846	0.565	0.583	0.603	0.608
Seq-aSum	0.984	0.928	0.787	0.672	0.717	0.637	0.569	0.555
Seq-aSum-VS	0.984	0.925	0.799	0.668	0.710	0.641	0.577	0.552
**LiMB** (*κ* = 0.90)								
Average no. branches	1.2	2.3	4.8	8.7	1.2	2.3	4.1	6.8
Average no. pared branches	1.0	1.0	1.1	1.1	1.0	1.0	1.1	1.1
wscore	0.981	0.919	0.725	0.589	0.722	0.654	0.579	0.542
pared wscore	0.984	0.917	0.728	0.582	0.714	0.642	0.572	0.526
**BUR** (*κ* = 0.95)								
Average no. branches	3.4	33.9	485	10098	1.5	6.1	58.7	985
Average no. pared branches	1.1	1.2	1.4	1.8	1.0	1.1	1.4	1.9
wscore	0.984	0.916	0.775	0.653	0.709	0.648	0.594	0.578
pared wscore	0.983	0.902	0.720	0.573	0.708	0.643	0.569	0.560
**BUR** (*κ* = 0.99)								
Average no. branches	1.4	2.6	5.8	16.5	1.1	1.5	2.4	5.2
Average no. pared branches	1.1	1.1	1.2	1.4	1.0	1.1	1.2	1.3
wscore	0.984	0.919	0.788	0.667	0.710	0.642	0.577	0.554
pared wscore	0.984	0.919	0.790	0.669	0.710	0.640	0.579	0.556

In the next situation, we have strong LD and causal RVs are associated in opposite directions. As in Basu and Pan [14], model selection has a sizable advantage over collapsing and random effect methods when there are few non-causal RVs present. However, as non-causal RVs are introduced in the model, model selection loses its advantage to SKAT while still maintaining superior advantage over the Sum test. The extension to model-averaging, though, appears to have superior performance to model selection when a *κ* of 0.95 is used. Due to strong LD, the non-causal RVs will have similar effects as the causal ones. Now moving one such variant with negative directional effect to the ‘-1’ category will be very similar to moving that variant to the ‘0’ category, since the other correlated RVs will still be in the ‘+1’ category, canceling the effect of this variant. Hence, an incorrect ‘0’ allocation could be assigned to this variant. Until all of the variants with negative directional effect are moved to the ‘-1’ category, we might not see much improvement in the likelihood. In this case, considering multiple allocations such as ‘-1’ and ‘0’ allocations for these variants would have better chance of finding the model that will significantly increase the likelihood of the data. Thus, BUR with *κ* = 0.95 presents a significant increase in power from Seq-aSum-VS by averaging over many models. We also can note that paring down to higher weighted models loses power in the case of strong LD. Additionally, the unpared version of BUR with *κ* = 0.95 only has slightly less power than SKAT when there are 8 or 12 non-causal RVs present. This power comparison suggests that these model-averaging methods could be quite useful when the RVs are in strong LD with few causal variants in opposite direction of association and in the presence of few non-causal variants.

#### Simulation 5: Mix of CVs and RVs

Here, we consider an analysis with a mixture of independent CVs and RVs. As recommended by [25], we have added in SKAT-C which gives uniform weight to CVs rather than the Beta(MAF; 1, 25) weight of SKAT which will severely downweight CVs. When CVs are mixed into a RV analysis, which is a usual scenario if you are scanning across a gene, the strength of contribution of CVs and RVs greatly influences which method will perform best. For Table 4, we first simulate 4 RVs with either shared common OR of 2 or two RVs with OR of 2 and the other two with OR of 1/2. We also simulated 3 moderately associated CVs of either OR = (1.2, 1.2, 1.2) or OR = (1.2, 1.2, 0.8). We then simulate 1, 5, 9, and 13 independent null CVs to study the impact of null variants on power. Because SKAT underweights these CVs, it suffers huge power loss compared to the other methods and as expected, SKAT-C performs much better than SKAT. SKAT would only perform well if mostly RVs contribute to disease risk [25]. SKAT-C is the top method when both CVs and RVs contribute to the risk but Seq-aSum-VS and BUR perform almost as well when there are a small number of null variants. Sum, as usual, suffers huge power loss in the presence of opposite directional effects. For the last situation, we make all of the RVs null and simulate 3 associated CVs with OR = (1.2, 1.2, 1.2). Unlike before, we can see that if only the CVs are associated, model selection and model-averaging performs well above the competitors, and BUR with *κ* = 0.95 shows significant improvement over Seq-aSum-VS when there are not many null CVs. For all of these situations, we see that Seq-aSum-VS and BUR do quite well especially if there is low number of null variants. This type of situation would be very common while scanning across a gene because variants in a window are likely to be in high LD and thus have a non-null effect.

**Table 4:** (*α* = 0.05) Analysis with a combination of RVs and CVs. Power in table based on 10000 replicates for each situation with a mix of common and rare variants. The CVs and RVs are all independent of each other.

RVs	OR = (2,2,2,2)				OR = (2, 2, 1/2, 1/2)				OR = (1, 1, 1, 1)			
Associated CVs	OR = (1.2,1.2,1.2)				OR = (1.2, 1.2, 0.8)				OR = (1.2,1.2,1.2)			
Null CVs	1	5	9	13	1	5	9	13	1	5	9	13
Sum	0.845	0.576	0.428	0.327	0.141	0.098	0.082	0.072	0.430	0.321	0.255	0.207
SKAT	0.496	0.501	0.488	0.490	0.404	0.391	0.390	0.387	0.227	0.169	0.145	0.119
SKAT-C	0.791	0.717	0.674	0.631	0.778	0.684	0.633	0.590	0.494	0.422	0.378	0.327
Seq-aSum	0.729	0.463	0.357	0.292	0.669	0.436	0.341	0.292	0.651	0.446	0.368	0.311
Seq-aSum-VS	0.760	0.523	0.432	0.333	0.672	0.449	0.379	0.304	0.681	0.465	0.412	0.329
**LiMB** (*κ* = 0.90)												
Average no. branches	1.5	2.3	3.9	8.3	1.7	3.2	6.4	12.6	1.4	1.8	2.7	4.7
Average no. pared branches	1.0	1.1	1.1	1.2	1.1	1.2	1.2	1.2	1.0	1.0	1.0	1.0
wscore	0.233	0.174	0.069	0.049	0.531	0.300	0.167	0.127	0.144	0.129	0.110	0.061
pared wscore	0.230	0.171	0.073	0.053	0.526	0.297	0.174	0.133	0.147	0.130	0.112	0.063
**BUR** (*κ* = 0.95)												
Average no. branches	8.0	27.8	161	1245	7.2	25.7	137	965	3.6	13.8	78.2	577
Average no. pared branches	2.0	2.5	3.2	4.3	1.9	2.6	3.5	4.9	1.1	1.3	1.7	2.4
wscore	0.766	0.515	0.412	0.316	0.677	0.451	0.371	0.304	0.692	0.466	0.398	0.324
pared wscore	0.761	0.527	0.429	0.329	0.668	0.451	0.374	0.304	0.690	0.481	0.415	0.332
**BUR** (*κ* = 0.99)												
Average no. branches	1.7	2.3	3.5	5.8	1.8	2.5	3.7	6.0	1.3	1.8	2.7	4.4
Average no. pared branches	1.4	1.6	1.9	2.3	1.6	1.9	2.2	2.7	1.1	1.1	1.3	1.5
wscore	0.761	0.524	0.431	0.333	0.672	0.449	0.377	0.304	0.683	0.466	0.412	0.329
pared wscore	0.760	0.525	0.431	0.334	0.670	0.447	0.376	0.305	0.682	0.469	0.412	0.331

### 3.4 Sanofi Data

Genomic intervals covering two genes that encode the endocannabinoid metabolic enzymes, FAAH and MGLL, were sequenced in 289 individuals of European ancestry using the Illumina GA sequencer [31]. Ancestry was determined using a panel of ancestry informative markers and individuals with an outlying genetic background were removed from the analysis. Sequencing was done using 36 base pair reads. The median coverage was 60X across the individuals sequenced. The program MAQ was used for alignment and variant calling, resulting in 1410 high quality single nucleotide variants (SNVs; 228 in the FAAH gene and 1182 in the MGLL gene) which were used for association analysis. The sequenced regions were captured using long range PCR and represented a total of 188,270 nucleotides. The 289 individuals included 147 normal controls (Body Mass Index (BMI) < 30) and 142 extremely obese cases (BMI > 40). Each region was analyzed separately with a sliding window of 1000 bp in length. The size of this sliding window was chosen to ensure a reasonably small number of SNVs being analyzed at one time. The number of variants included in any window of either gene varies from 2-25 but about 90% included 5-15 SNVs. There were both common and rare (MAF ≤ 0.01) variants in the windows. Table 5 shows the distribution of the RVs and CVs in the reported windows. When available, we used the asymptotic distribution to calculate p-values. Otherwise, 1000 permutations were used to calculate p-values at each sliding window. Due to the poor performance of LiMB in the simulations, we have dropped it from the real data results. Because we have a mix of RVs and CVs, we have added SKAT-C which upweights CVs as compared to SKAT [25]. At each window, we recorded the minimum p-value of the competing methods. An additional 9000 permutations were performed for the most significant windows of each gene. To measure LD, we use the D’ statistic [32]. The mean LD in each of the sliding windows was moderate with *D*′ = 0.448 and 0.449 in the FAAH and MGLL genes, respectively. *D*′ ranged from 0.012 to 0.9996 for all sliding windows.

**Fig 3:**
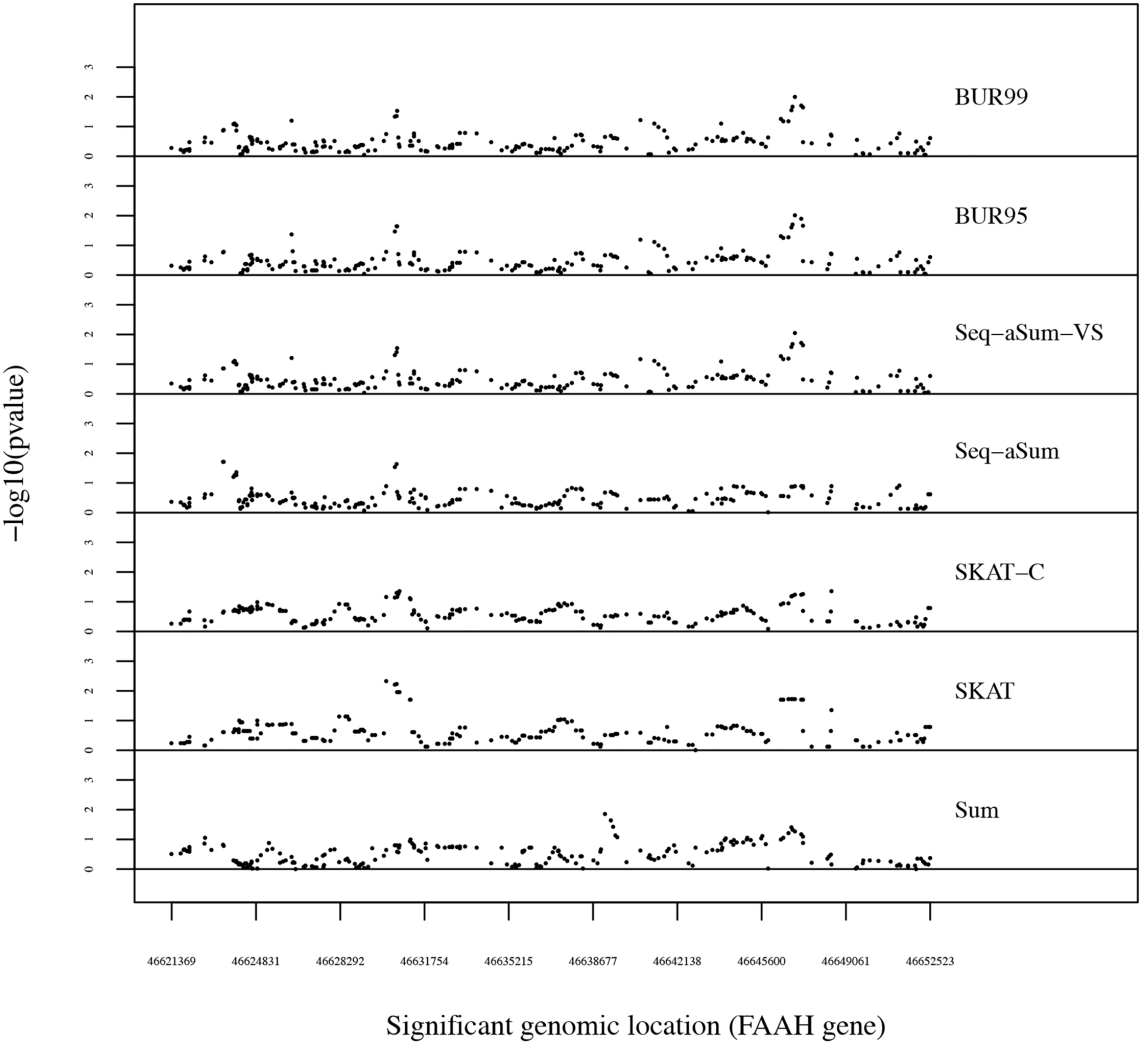
A sliding window analysis of FAAH gene for each method from top to bottom: BUR with *κ* = 0.95 (BUR95), BUR with *κ* = 0.99 (BUR99), Seq-aSum-VS, and Seq-aSum, SKAT-C, SKAT, and Sum. A window size of 1000 bp is used. The −log_10_(p-value) for each window is plotted on the y-axis. The beginning genomic location of each window is plotted across the x-axis. Each point represents the −log_10_(p-value) of one window.

**Fig 4:**
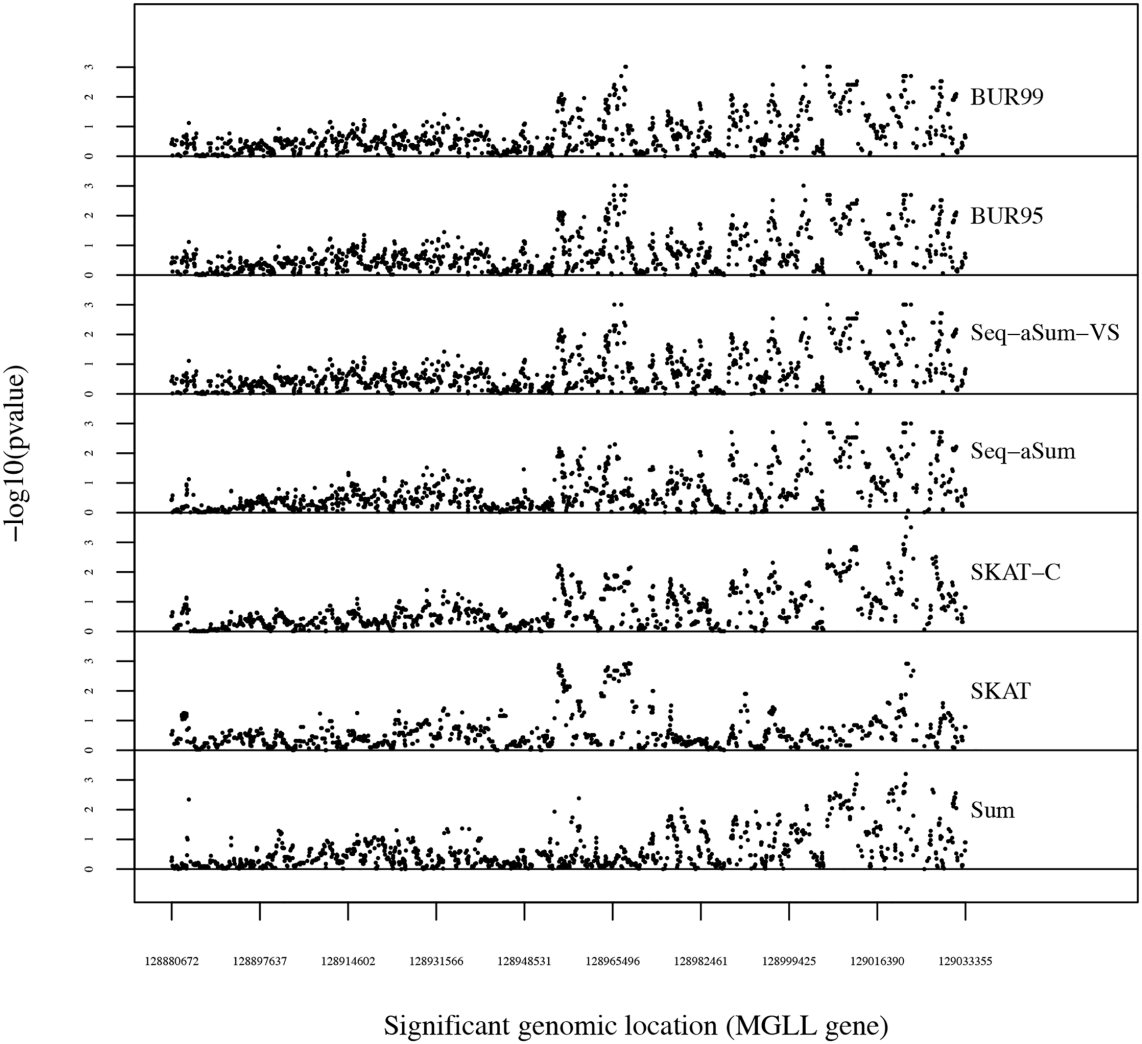
A sliding window analysis of MGLL gene for each method from top to bottom: BUR with *κ* = 0.95 (BUR95), BUR with *κ* = 0.99 (BUR99), Seq-aSum-VS, and Seq-aSum, SKAT-C, SKAT, and Sum. A window size of 1000 bp is used. The −log_10_(p-value) for each window is plotted on the y-axis. The beginning genomic location of each window is plotted across the x-axis. Each point represents the −log_10_(p-value) of one window.

Figs 3 and 4 plot the −log_10_(p-values) of each window as it slides across FAAH and MGLL, respectively. The most significant windows of the 228 in the FAAH gene and the ten most significant windows of the 1182 in the MGLL gene are reported in Table 5 with bolded p-values for the best p-value in each window. We also denote the order of the sliding window and its starting genomic location.

**Table 5:** Top −log_10_(p-values) with window starting genomic location and the order of the frame for data analysis of both genes. BUR95 represents the BUR (*κ* = 0.95) approach and BUR99 represents the BUR (*κ* = 0.99) approach. The top p-value among these approaches is in bold for each window and the number of branches averaged over is listed in parentheses.

			Total					Model selection		Model-averaging	
Gene	Start Pos.	Window	RV	CV	Sum	SKAT	SKAT-C	Seq-aSum	Seq-aSum-VS	BUR95	BUR99
**FAAH**	46630186	85	3	6	0.647	**2.321**	1.159	0.872	0.738	0.756 (4)	0.736 (1)
	46630533	86	4	10	0.798	**2.213**	1.144	1.479	1.279	1.466 (2)	1.318 (2)
	46630611	87	4	10	0.787	**2.218**	1.302	1.503	1.372	1.611 (3)	1.326 (2)
	46630632	88	4	9	0.590	**1.952**	1.157	0.688	1.476	1.664 (1)	1.479 (1)
	46630673	89	3	9	0.561	**1.947**	1.260	0.580	0.647	0.705 (3)	0.624 (2)
	46630715	90	3	8	0.719	**1.947**	1.329	0.468	0.393	0.426 (9)	0.393 (1)
	46630718	91	3	7	0.788	**1.947**	1.347	0.500	0.304	0.355 (18)	0.317 (2)
	46639163	153	4	3	**1.854**	0.511	0.505	0.695	0.680	0.691 (1)	0.68 (1)
	46646969	199	4	2	1.265	1.709	1.230	0.859	1.880	**1.932 (1)**	1.88 (1)
	46647235	200	3	2	1.175	1.706	1.243	0.851	1.708	**1.785 (1)**	1.708 (1)
**MGLL**	128967903	966	8	3	0.583	**2.887**	1.626	0.730	2.387	2.796 (3)	2.432 (1)
	128967981	967	7	4	0.382	2.884	1.639	**3.301**	3.097	3.097 (2)	3.097 (1)
	128968059	968	6	5	0.342	2.785	1.662	1.146	2.620	**2.854 (1)**	2.62 (1)
	129002169	1228	0	5	1.165	0.556	1.107	2.538	3.000	**3.046 (2)**	3.000 (1)
	129002562	1229	1	5	1.205	0.561	1.195	2.658	2.482	**2.824 (6)**	2.469 (1)
	129006765	1251	5	2	1.438	0.104	2.245	**3.097**	2.959	2.569 (10)	2.959 (1)
	129007295	1255	2	2	2.317	0.750	**2.721**	2.678	2.658	2.569 (4)	2.658 (1)
	129012512	1277	3	2	**3.195**	0.839	2.262	2.699	2.553	2.523 (4)	2.553 (1)
	129021383	1324	5	4	2.562	1.261	**2.929**	2.699	2.398	2.399 (12)	2.524 (2)
	129021891	1329	1	3	3.191	1.882	**3.196**	2.854	2.602	2.569 (2)	2.602 (1)
	129021962	1330	1	3	1.245	2.913	**3.841**	1.903	1.572	1.582 (1)	1.572 (1)
	129022269	1331	0	3	1.338	2.914	**4.066**	2.056	1.791	1.81 (1)	1.791 (1)
	129022900	1332	2	2	0.988	2.503	**3.497**	3.000	2.959	2.959 (3)	2.959 (2)

Like in previous analyses [31], the analysis shows little significant association of the FAAH gene with obesity in Fig 3. None of the p-values come close to the multiple comparisons level of significance of 4.06 [31]. We can see that the Sum test and model selection without variable selection drown out the faint signals shown by the other methods. The BUR approaches perform almost identically to Seq-aSum-VS.

The MGLL gene does show some suggestion of consistency of a signal in the rightmost region in Fig 4. BUR with *κ* = 0.95 seems to amplify the signal in this area as compared to Seq-aSum-VS. Also, besides the middle-most region, SKAT appears to lack most of the signal shown by the other methods. Seq-aSum-VS and BUR seem to capture both the middle and right-most feature of the MGLL gene whereas the other methods only capture one or the other. SKAT-C also captures these regions, but with the exception of the few windows shown in Table 5, Seq-aSum-VS and BUR show more significance. From Table 4, we might hypothesize that the right-most region has some moderate CV effects which SKAT fails to detect but SKAT-C, model selection, and model-averaging do detect. SKAT performs best when RVs contribute most to the risk such as in the windows of FAAH. Meanwhile, SKAT-C performs well when CVs and RVs are both contributing as they may be the case in the last few windows shown in Table 5. Model selection and BUR do quite well if CVs contribute to the risk, and from our simulations, they perform best when CVs contribute to most of the risk as they may in windows 1228 and 1229.

By looking at the top p-values in Table 5, we can assess the potential gains that model-averaging has over model selection. First of all, because the null distribution of model-averaging is stochastically smaller than model selection as we decrease *κ*, we can see that even when only one path is selected for BUR with *κ* = 0.95, it usually obtains a lower p-value than model selection and its close counterpart BUR with *κ* = 0.99. Also, out of the 16 top windows that BUR with *κ* = 0.95 has multiple paths averaged over, 10 of them produce a better p-value than when only one path is chosen by Seq-aSum-VS.

## Discussion

In this paper we have studied the performance of several weighted score tests implementing model-averaging approaches and compared them to their competitors in detection of rare variants. It has been well documented [14] that no method is uniformly most powerful. Each method is very dependent on the underlying unknown true model. We have shown through simulation that each method has situations where it performs better than its competitors. However, through our simulations and the real data analysis we found that the Seq-aSum-VS and BUR approaches maintain reasonable power in almost all situations and never suffer huge power loss unlike the other methods, particularly when we have both CVs and RVs in the analysis and the CVs strongly contribute to disease risk. In fact, model selection and BUR were some of the top methods in all simulations when there were a low number of null variants. This situation would be very typical while scanning across a causal gene because variants in a window are likely to be in high LD with the causal variant and thus all have a non-null effect. We have focused on the comparison of model-averaging with model selection approaches. While the advantages of model-averaging have been well documented in the prediction literature [27], we studied the advantage of model-averaging over model selection when our purpose is for inference. As shown in simulation studies and real data analysis, model-averaging over a limited number of models showed a power gain over model selection, but the power gain was not substantial in most simulation setups. One possible explanation could be that the model selection approach already implements a dimension reduction strategy which requires estimation of only three parameters for each model. Due to the small number of parameters, the uncertainty in model selection decreases and the advantage of model-averaging over model selection becomes less significant.

The model-averaging approach was proposed to reduce the dependency of the model selection approaches such as Seq-aSum and Seq-aSum-VS on the sequential selection of the SNPs. The performance of a model selection approach would depend on the order at which the SNPs were selected sequentially. A model-averaging approach, on the other hand, reduces this order dependency. In addition to this reduction of order dependency, we saw that averaging over more models can present a gain in power over one model, particularly when variants are in strong LD and when there is a mix of causal and protective RVs. We also saw in our simulations and possibly the real data analysis that BUR with *κ* = 0.95 had significant gains over model selection when CVs strongly contribute to the risk with only a small number of null variants. So while model selection was presented as a good middle approach for any alternative disease model in Basu and Pan [14], model-averaging is perhaps more advantageous because it performs as well or better depending on the truth.

If covariates were to be added, permutation of the outcomes would no longer suffice if the goal is to test the genetic effect. Instead, one could fit a model with only the covariates and then use a parametric bootstrap using the estimated covariate effects to simulate the same amount of datasets that you would use in a permutation [33]. We then proceed in a similar fashion as a permutation approach where we perform model-averaging on the simulated set and compare our test statistic to the bootstrapped test statistics.

In general, the BUR approach with 0.95 cutoff performed better than the BUR approach with 0.99 cutoff, which indicates that there is a clear benefit from averaging over more models since it accounts for the model uncertainty. When too many models are averaged over with independent RVs, the BUR approach with 0.95 cutoff is still better but we need to pare down the branches. On the other hand, one big limitation of model-averaging is the number of models you average over. It became too computationally intensive once we considered more than 20 variants. Hence, model-averaging has an advantage over model selection when we consider a small to moderate number of variants. From the simulations, we would recommend using the BUR approach with *κ* = 0.95 in order to search a wide array of models. If the variants in the SNV-set are independent or weakly correlated, we would also recommend paring down to only the top models in order to reduce the number of models to average over. Use of this recommended application of our proposed model-averaging is illustrated in the real data section. A sliding window with 5-20 variants could give us optimal performance of the model-averaging approach. In the future, we intend to compare this model-averaging approach with a model-averaging approach with distinct parameters for each directional effect in the BUR approach, while undergoing variable selection. We believe there would be substantial power gain over this reduced model with same effect size for both directions.
